# How Close We Are to Achieving Commercially Viable Large-Scale Photobiological Hydrogen Production by Cyanobacteria: A Review of the Biological Aspects

**DOI:** 10.3390/life5010997

**Published:** 2015-03-18

**Authors:** Hidehiro Sakurai, Hajime Masukawa, Masaharu Kitashima, Kazuhito Inoue

**Affiliations:** 1 Research Institute for Photobiological Hydrogen Production, Kanagawa University, Tsuchiya, Hiratsuka, Kanagawa 259-1293, Japan; 2 The OCU Advanced Research Institute for Natural Science and Technology (OCARINA), Osaka City University, 3-3-138 Sugimoto, Sumiyoshi-ku, Osaka 558-8585, Japan; E-Mail: masukawa@ocarina.osaka-cu.ac.jp; 3 Department of Biological Sciences, Kanagawa University, Tsuchiya, Hiratsuka, Kanagawa 259-1293, Japan; E-Mails: pt125536zy@kanagawa-u.ac.jp (M.K.); inouek01@kanagawa-u.ac.jp (K.I.)

**Keywords:** cyanobacteria, hydrogen, photosynthesis, nitrogenase, heterocyst, renewable energy, mariculture

## Abstract

Photobiological production of H_2_ by cyanobacteria is considered to be an ideal source of renewable energy because the inputs, water and sunlight, are abundant. The products of photobiological systems are H_2_ and O_2_; the H_2_ can be used as the energy source of fuel cells, *etc.*, which generate electricity at high efficiencies and minimal pollution, as the waste product is H_2_O. Overall, production of commercially viable algal fuels in any form, including biomass and biodiesel, is challenging, and the very few systems that are operational have yet to be evaluated. In this paper we will: briefly review some of the necessary conditions for economical production, summarize the reports of photobiological H_2_ production by cyanobacteria, present our schemes for future production, and discuss the necessity for further progress in the research needed to achieve commercially viable large-scale H_2_ production.

## 1. Introduction

### 1.1. Global Climate Change

**Figure 1:**
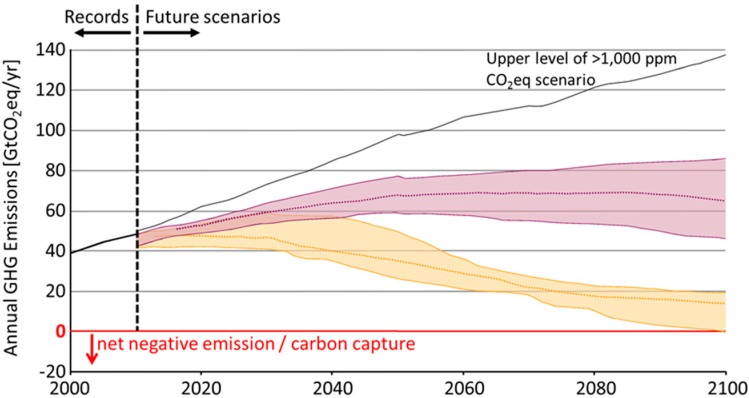
Some scenarios for annual greenhouse gas (GHG) emission, UN IPCC. Due to uncertainty regarding future emissions and prediction of the outcome, probable ranges are shown for each stabilization targets. █ Stabilization at 530–580 and █ 720–1000 ppm CO_2_-eq respectively in 2100. The upper level of the probable range of >1000 ppm CO_2_-eq line in IPCC scenario is also shown. (Adapted from Figure SPM4, IPCC, 2014: Summary for Policy Makers, the Climate Change 2014 [1]).

The concentration of atmospheric CO_2_ has been increasing since the era of the industrial revolution when it was estimated to be 270–280 ppm initially, rising to current levels of about 400 ppm. According to the fifth assessment report of the UN IPCC (United Nations Intergovernmental Panel on Climate Change) [1,2], the greatest contribution to the global increase in greenhouse gases comes from CO_2_ emitted by burning fossil fuels (65%) and land use changes (deforestation) (11%), followed by methane (16%), N_2_O (6%) and fluorocarbons, *etc.* (2%). Currently, the amounts of greenhouse gases emitted have continued to rise due to the increases in human activities and population growth. If we are able to stabilize the atmospheric greenhouse gases at 530–580 ppm-CO_2_ equivalent (approximately twice that of the pre-industrial level), one of the ICPP scenarios predicts that we will need to reduce global greenhouse gas emissions by 19%–47% in 2050 and 59%–81% in 2100 (relative to 2010 emissions) (Figure 1). Some scenarios predict that we will need to go beyond reduction strategies and achieve “negative emissions” by CO_2_ sequestration, *etc.* Economically advanced countries will likely be required to reduce emissions the most, perhaps by as much as 80% compared to present levels. Even if we succeed in stabilizing greenhouse gases at the level of 530–580 ppm CO_2_ equivalent, the global average temperature will rise around 2.0–2.2 °C relative to the 1850–1900 temperatures. Stabilizing emissions at a less-stringent level of 720–1000 ppm-CO_2_ equivalent, would still require substantial emissions reduction from current, largely unregulated levels, and result in the average temperature rising around 3.1–3.7 °C relative to the 1850–1900 temperatures. From all of these considerations, it is evident that we urgently need viable renewable energy technologies as part of a global strategy to drastically reduce CO_2_ emissions from burning fossil fuels and thus avert potentially catastrophic climate change.

### 1.2. Prospects for Large-Scale Algal Biofuel Production

We are currently consuming energy at an unsustainably high rate, with much of the consumption (about 82%) coming from fossil fuels (Table 1) (International Energy Agency (IEA), IEA 2014) [3]. Land-based production of biofuels has been proposed to meet at least some of our energy needs. One method for assessing the energy potential of land-based production systems is to consider total primary energy consumption in the context of the nutritional energy consumption largely derived from current land-based crop systems. In terms of total primary energy consumption, on a world-wide average, people consume about 25 times their nutritional energy (20 times, compared with the fossil fuel-derived energy) (Table 1): people in US 136, Germany 76, Japan 70, China 43 times, respectively (IEA Energy Indicators 2012 in (IEA Energy Outlook 2014)) [4]. From this comparison, it is evident that we cannot expect much more additional energy from land-based agricultural crops, such as sugar cane and corn. Accordingly, there are growing interests in the development and production of biofuels, such as biomass, biodiesel, and H_2_, using a variety of microalgae grown on lands that are not suitable for agricultural production [5,6,7,8,9]. However, commercially viable large-scale fuel production from microalgae has yet to be realized.

**Table 1:** Solar energy and human energy consumption.

	Energy Intensity and Quantity	Ratios			Refs./Remarks
Solar energy received on Earth surface					
Intensity	165 W·m^−2^ (1450 kWh·m^−2^·yr^−1^)				[10]
Total energy	2,660,000 × 10^18^ J·yr^−1^	4750	5800	440,000	[10]
Photosynthetically active radiation	1,200,000 × 10^18^ J·yr^−1^				45% of the total
Human social energy consumption					
Total primary energy supply	560 × 10^18^ J·yr^−1^	1	1.22	25	[3]
Fossil fuel consumption	460 × 10^18^ J·yr^−1^	0.82	1	21	[3]
Human digestive energy intake	22 × 10^18^ J·yr^−1^	0.039	0.048	1	Population: 7.2 × 10^9^ 2000 kcal·capita^−1^·day^−1^

### 1.3. Examples of Policies to Promote the Development of Renewable Energy Technologies

#### 1.3.1. Feed-In Tariff Scheme in Germany

Feed-in electricity tariffs were introduced in Germany in 2004 to encourage the use of new energy technologies, such as wind, hydro- and geo-thermal power, biomass, and solar photovoltaics. Feed-in tariffs are a policy employed by governments to encourage investment in renewable energy technologies by providing them a fee (or “tariff”) above the retail rate of electricity. Feed-in tariffs provide long-term investment security to renewable energy producers and are typically based on the costs of specific technology. Wind power, for instance, costs less to produce than solar PV or tidal powers, so the tariff awarded is correspondingly lower. In a Wikipedia entry [11], the German feed-in tariff system is described as one of the most efficient and effective support schemes in the world for promoting renewable electricity. As of August 2014, a revised Renewable Energy Sources Act (EEG 2014, also called EEG 2.0) was implemented in Germany, with some modifications to the feed-in tariffs. The aim is to meet Germany’s renewable energy goals of 40% to 45% of electricity consumption in 2025 and 55% to 60% in 2035 [12].

For an international comparison of the price of energy and feed-in tariff, the following currency exchange rates (approximate average currency exchange rates in 2014) were assumed in this paper: 1 Euro = 132 US¢, and 100 Japanese Yen = 94 US¢. The German feed-in tariffs in August of 2014 are: 4.62 US¢/kWh for hydropower facilities up to 50 MW, 16.5 ¢/kWh for those up to 500 kWh, 16.4 ¢/kWh for solar installations on buildings up to 10 kW and 25.6 ¢/kWh for offshore wind [12]. The tariffs for photovoltanics in 2014 decreased by about 80% compared with those in 2004, the starting year of the German program, demonstrating the effectiveness of the policy strategy.

#### 1.3.2. Feed-In Tariff Scheme in Japan [13]

The government of Japan introduced feed-in tariffs in 2012. In April 2014, the purchase prices (excluding the tax, with the purchase period of 10 or 20 years at fixed prices) were: 34.8 ¢/kWh for photovoltanics up to 10 kWh, 30.1 ¢/kWh for those 10 kW or more, 20.7 ¢/kWh for land-based wind power, 33.8 ¢/kWh for offshore wind power, and 13.2 ¢/kWh for medium hydropower. Because the prices offered by the government were so favorable to investors, in southern Japan, Kyushu Electric Power Co. had to suspend receipt of applications from large-sized renewable-energy producers (photovoltanics) wishing to access the company’s grid while it reviews how much more clean energy it’s capable of handling on sunny days.

#### 1.3.3. Estimated Cost of Electricity in the USA

According to the 2012 estimates by the US Energy Information Administration (eia), the levelized cost of electricity in ¢/kWh for plants entering service in 2019 are: conventional coal 9.56 (no subsidy), geothermal 4.79 (4.45, with subsidy), solar photovoltanic 13.0 (11.86 with subsidy), *etc.* [14]. These estimates illustrate that for the US, coal continues to be a relatively cheap source of electricity even though burning coal emits a large amount of CO_2_ into the atmosphere. In order to decrease CO_2_ emissions from coal, the integrated coal-gasification combined cycle (IGCC) (estimated to be 11.59 ¢/kWh) and IGCC with carbon control and sequestration (CCS) systems have been proposed (estimated to be 14.74 ¢/kWh). The CO_2_ captured in these systems is transported to a storage site, normally an underground geological formation. The effectiveness of CCS, and its long-term effects on the environment need to be carefully assessed.

#### 1.3.4. Possible Merit of H_2_ as Motor Fuels

The next-generation of fuel-efficient cars is entering the consumer market. Toyota recently announced the commercialization of hydrogen fuel-cell vehicles called “Mirai” in December 2014 [15]. According to Toyota, using measurements based on the Japanese JC08 test cycle, a cruising range of approximately 700 km can be achieved when fueled with H_2_ at 70 MPa. In comparison, the cruising ranges of electric cars might be around 100–200 km per full battery charge. In the same month, several Japanese gas companies announced opening commercial hydrogen refueling stations with the introductory prices of H_2_ (subsidized by the government) ranging from 9.4 to 10.3 US$ (1000–1100 Japanese Yen) per kg, or 23.8–26.1 ¢/kWh [16]. Because the energy efficiency of fuel cell cars is better than internal combustion engine cars, it is estimated that H_2_ fuel will be competitive with gasoline 1.22–1.60 US$/L (retail price of about 13.9–18.3 ¢/kWh including tax, calculated on an energy density of 8.76 kWh/L).

## 2. Outline of the Biological Processes of Photobiological H_2_ Production by Heterocyst-Forming Cyanobacteria

Photobiological production of biomass by photosynthetic organisms (trees, grasses, algae, *etc.*) is considered one of the better candidates for large-scale, renewable energy because the amount of solar energy is almost infinite (Table 1). Photobiological H_2_ production by cyanobacteria is one of the options, and this review briefly discusses nitrogenase (N_2_ase)-based photobiological H_2_ production by cyanobacteria because it is the focus of our current studies. In doing so, we do not intend to supplant other systems, such as hydrogenase (H_2_ase)-based photobiological H_2_ production by various algae including cyanobacteria, as well as biofuel production (e.g., biomass, biodiesel) by various photosynthetic microorganisms.

Some strains of cyanobacteria possessing N_2_ase are good candidates for optimization of photobiological H_2_ production [17,18,19,20,21]. N_2_ase catalyzes the following reaction under optimal conditions for N_2_ fixation:

N_2_ + 8 e^−^ + 8 H^+^ + 16 ATP → H_2_ + 2 NH_3_ + 16 (ADP + P_i_) (1)

whereas, in the absence of N_2_ (e.g., under Ar), all e^−^s are allocated to H_2_ production:

2 e^−^ + 2 H^+^ + 4 ATP → H_2_ + 4 (ADP + P_i_) (2)

The above reactions catalyzed by N_2_ase are essentially irreversible. N_2_ase is extremely O_2_ sensitive and quickly inactivated by O_2_. In order to reconcile the activities of the O_2_-sensitive N_2_ase with O_2_-evolving photosynthesis, cyanobacteria have evolved various means to address to the physiological challenges [22]. There are three basic groups. In Group 1, the two processes are separated spatially as found in heterocystous filamentous types (e.g., *Anabaena, Nostoc, Calothrix*). In Group 2, the processes are separated temporally by circadian rhythm as found in non-heterocystous unicellular and filamentous types (e.g., *Cyanothece, Lyngbya*). In Group 3, other non-heterocystous filamentous types (e.g., *Trichodesmium*) seem to sporadically perform the two processes: some of the cells have ordinary O_2_-evolving photosynthesis activity, while the others temporally cease photosynthesis and fix N_2_ [23].

**Figure 2:**
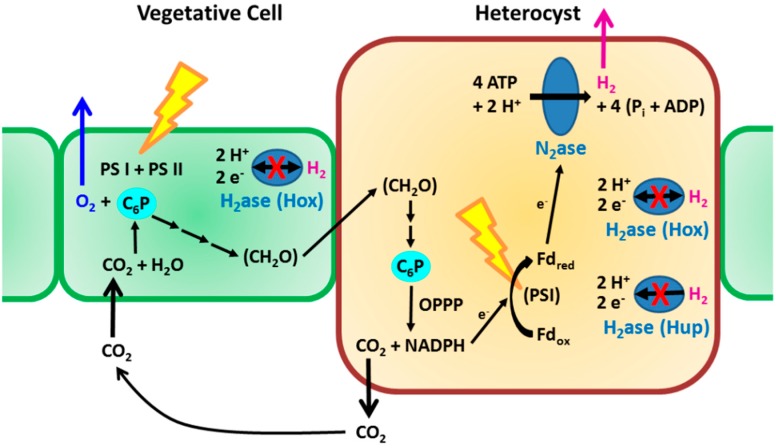
Outline of H_2_-related metabolic routes in heterocyst-forming cyanobacteria. Vegetative cells synthesize saccharides (CH_2_O) by ordinary photosynthesis with accompanying evolution of O_2_ and uptake of CO_2_. Heterocysts receive the saccharides, and use them (accompanied by CO_2_ evolution) as the sources of e^−^ for N_2_ase reaction. For efficient net production of H_2_, H_2_ase(s) (uptake H_2_ase Hup and bidirectional H_2_ase Hox) have been inactivated. C_6_P: hexose phosphate, Fd_ox_ and Fd_red_: ferredoxin oxidized and reduced respectively, OPPP: oxidative pentose phosphate pathway, PSI and PSII: photosystem I and II, respectively (adapted from [21] with modification).

In this review, we will discuss mainly Group 1 cyanobacteria, as these strains are the focus of our research and because they are the most extensively studied among the three groups with respect to physiology, molecular biology, *etc.* [24,25,26]. The cells are organized as filaments, with the majority of the cells (called vegetative cells) synthesizing organic compounds by ordinary photosynthesis. Under combined-nitrogen deficiency a few of the cells develop into heterocysts, cells specialized for nitrogen fixation. Heterocysts lacking photosystem II activity, have increased respiration and are surrounded by a thick cell envelope that impedes the entry of O_2_, thus providing a micro-oxic environment to protect the N_2_ase from inactivation by O_2_. They receive saccharides from neighboring vegetative cells and the saccharides are then used as the electron donors for the N_2_ase reaction (Figure 2). Within heterocysts, photosystem I reduces low-potential ferredoxin and/or flavodoxin and contributes to the generation of ATP through photophosphorylation. The fixed nitrogen is converted to glutamine, which is transported to vegetative cells. In this manner, heterocystous cyanobacteria are able to simultaneously perform O_2_-evolving photosynthesis and the O_2_-labile N_2_ase reaction.

Many of these types of cyanobacterial strains have the uptake hydrogenase (Hup) and some (but not all) of them also have the bidirectional hydrogenase (Hox) [27,28,29]. The former H_2_ase reabsorbs H_2_ produced by N_2_ase and thus the presence of Hup activity can limit the net production of H_2_ by the N_2_ase. There has been a report of an environmental isolate of the non-heterocystous *Oscillatoria* sp. strain Miami BG7 that lacks Hup activity and is able to accumulate H_2_ in the presence of photosynthetically evolved O_2_ [30]. When Hup activity was eliminated by molecular genetic techniques [28,31,32,33,34,35,36] or chemical mutagenesis [37], the resulting cyanobacterial mutants showed enhanced H_2_ production activity. Since in contrast to H_2_ase activity, N_2_ase catalyzes an essentially unidirectional H_2_ production and thus does not consume H_2_, cyanobacterial mutants lacking Hup activity are able to accumulate H_2_ to about 7%–30% (v/v) in the presence of O_2_ evolved [35,38].

In the unicellular diazotrophic cyanobacterium *Cyanothece* sp. strain PCC 7822, the N_2_ase and H_2_ production activities are inactivated following disruption of the *hupL* gene (encoding a large subunit of Hup) via molecular genetic techniques, indicating that Hup protects N_2_ase from oxygen toxicity by removing O_2_ in this strain [39].

Although Hup may play such a role, the degree of its N_2_ase protection may differ among strains, and we can selectively use the strains (either heterocystous or unicellular) in which the negative effects of elimination of Hup on net H_2_ production are low.

## 3. Some Lessons from Studies of the Economics of Large-Scale Algal Fuel Production

### 3.1. Benemann’s Critical View of Algal Biofuel Production

Benemann has been studying photobiological biofuel production by various algae, since the first discovery of N_2_ase-based photobiological H_2_ production by cyanobacteria [40]. He critically examined the viability of microalgae for the production of gaseous fuels, specifically H_2_ and CH_4_, and assessed various proposed schemes on the basis of their technological and economic feasibility. In 2004, he summarized his opinion [41] as: “Processes for the production of gaseous fuels, H_2_ and CH_4_, using microalgae culture have been studied from a scientific perspective for several decades, and the practical cultures have been studied relatively recently. The lack of practical results in H_2_ and CH_4_ production cannot be ascribed to limited R&D funding. The only present practical application is the harvesting of algal biomass from wastewater treatment ponds by chemical flocculation, followed by the anaerobic digestion of the algal biomass. Overall, costs of such a process were estimated at about $10 per GJ of H_2_ (3.6 ¢/kWh).” Benemann’s conceptual steps for a techno-economic analysis consisted of: (1) Production in open ponds (costing $7 m^−2^, 0.5 km^2^ in area in some of his analyses), at a solar efficiency of 10%, of a nitrogen-limited biomass (that accumulates large amounts of storage carbohydrates); (2) Concentration of the biomass from the ponds in a settling pond; (3) Anaerobic dark fermentation to yield 4 H_2_/glucose stored in the algal cells, plus two acetate moles; and (4) Reaction in a photobioreactor in which the algal cells would be converted from the two acetate moles to eight moles of H_2_. The cost of the process was estimated at about $10 per·GJ^−1^ of H_2_, (3.6 ¢/kWh) with the photobioreactor comprising about half of the total cost. In our opinion, a photosynthesis efficiency of 10%, required for economical production, is very difficult to attain even if an algal H_2_ase serving as a highly efficient H_2_ catalyst is used.

### 3.2. Life-Cycle Analysis (LCA) of the Energy Requirements for Algal Fuel Production

Clarens *et al.* [42] made LCA analyses of biofuel production by microalgae based on published records (first-generation algae production), and found that algae production systems release more CO_2_ to the atmosphere than is taken up during growth of the biomass. Their schemes are: (1) Growth of algae in open ponds using a raceway configuration, with slow-moving paddle wheels to aerate and circulate the algae growth medium; (2) fertilizers are added as water is pumped in to or out of (for harvesting) the ponds; and (3) CO_2_ is bubbled into the ponds. The result of the analyses for a production site in California, for example, indicated that for producing 1.00 unit of energy, about 1.18 unit of energy is consumed; nutrients: 0.49 unit (about 0.2 unit for urea), CO_2_-derived: 0.35 unit, direct: 0.04 unit, and others (water, *etc.*): 0.29 unit. They pointed out that in addition to anticipated improvements in algal productivity (next-generation production), the uses of flue gas (from coal fired plants) and municipal wastewater (to reduce nutrient energy costs) could lead to a net-positive bioenergy production by microalgae in these systems.

### 3.3. Life-Cycle Analysis (LCA) of the Costs for Large-Scale Microalgal Production

A comparative LCA study of the potential environmental impact and economic viability of producing biodiesel from microalgae grown in large virtual ponds (4 km^2^) of coastal Australian land were made [43]. In terms of GHG (greenhouse gas) emissions, algae GHG (−27.6 to 18.2 (CO_2_-unit)) compare very favorably with canola (35.9) and ultra-low sulfur (ULS) diesel from oil (81.2). Costs are not so favorable, with algae ranging from 2.2 to 4.8 (cost-unit), compared with canola (4.2) and ULS diesel (3.8). The large footprint of algae cultivation is driven predominantly by upstream factors, such as the demands for CO_2_ and fertilizer. To reduce these factors, flue gas and, to a greater extent, municipal waste-water could be used to offset the economic and environmental burdens associated with algae, highlighting the need for a high production rate to make algal biodiesel economically attractive.

## 4. Our Scheme for Large-Scale Photobiological H_2_ Production by Mariculture-Raised Cyanobacteria

We briefly discuss several issues for achieving commercially viable large-scale photobiological H_2_ production by cyanobacteria. Photobiological H_2_ production is considered to be one of the better candidates for renewable energy production because H_2_ pollutes environment minimally both in production as well as in utilization stages, and is relatively easily storable and transportable.

**Figure 3:**
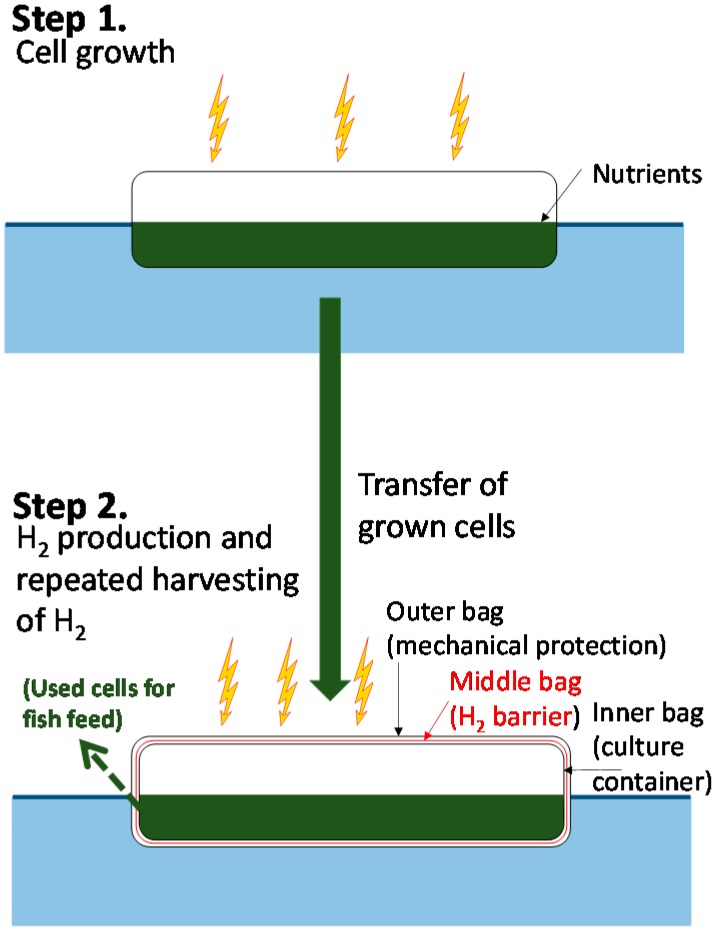
Outline of H_2_ production by mariculture-raised cyanobacteria. **Step 1:** Cell growth in a transparent plastic bag floating on the sea surface. The bioreactor is filled with air containing CO_2_; **Step 2**: H_2_ production in a photobioreactor composed of three bags (at least one layer is a H_2_ gas barrier membrane) floating on the sea surface. The spent cells can be used as fish feed [48].

**Figure 4:**
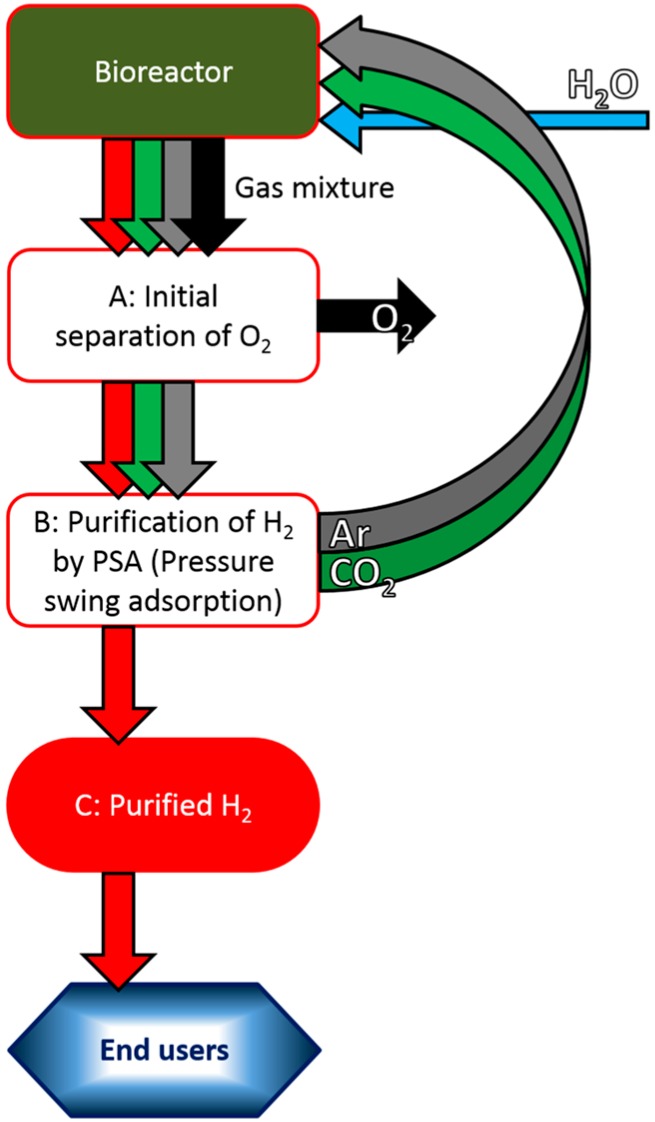
Outline of photobiological H_2_ production by mariculture-raised cyanobacteria and delivery of purified H_2_ to end-users (possible scheme). The fully grown cells (Step 1, Figure 3) are transferred to the photobioreactor (Step 2, Figure 3), which is filled with Ar and CO_2_ (+ trace amount of N_2_, not shown) and allowed accumulation of the produced gases (H_2_ and O_2_). (**A**) Initial separation of O_2_ from the gas mixture; (**B)** Further purification of H_2_ by PSA (pressure-swing adsorption), and the removed CO_2_ and Ar are recycled to the bioreactor. H_2_O consumed for H_2_ production is replenished; (**C)** Purified H_2_ is processed for transportation to end-users (for details, see text).

We will first describe an outline of our conceptual scheme [44] for large-scale photobiological H_2_ production by mariculture-raised cyanobacteria. We will then discuss some of the key issues in greater detail. Our scheme is: (1) Production of H_2_ in large plastic bioreactors consisting of several layers of plastic film; (2) repeated harvesting of crude H_2_; (3) initial separation of H_2_ from O_2_ possibly by gas-selective permeability membranes on factory ships followed by further purification of H_2_ by pressure-swing adsorption (PSA); and (4) compression or transformation to a form suitable for transportation by ship and storage (Figure 3 and Figure 4).

**Table 2:** Expected sales of photobiologically produced H_2_.

Energy Conversion Efficiency (%)	Produced H_2_ (kWh·m^−2^·yr^−1^)	Energy Recovery (Ratio)	Purified H_2_ (kWh·m^−2^·yr^−1^)	**H_2_ Total Sale (cents m^−2^·yr^−1^**)			
				Selling Price (cents·kWh^−1^)			
				10	20	30	40
1	15	0.3	4.5	**45**	**90**	**135**	**180**
1	15	0.5	7.5	**75**	**150**	**225**	**300**
1	15	0.7	10.5	**105**	**210**	**315**	**420**
2	30	0.3	9	**90**	**180**	**270**	**360**
2	30	0.5	15	**150**	**300**	**450**	**600**
2	30	0.7	21	**210**	**420**	**630**	**840**
3	45	0.3	9	**135**	**270**	**405**	**540**
3	45	0.5	15	**225**	**450**	**675**	**900**
3	45	0.7	21	**315**	**630**	**945**	**1260**

The Δ*H* of 1 kg crude oil (Oeq) is 41.9 MJ or 11.6 kWh (HHV, higher heating value: the product water is condensed liquid). The Δ*H* of 1 m^3^ (STP) H_2_ is 12.8 MJ, 3.56 kWh, or 0.30 kg·Oeq (Oil equivalent) and that of 1kg of H_2_ is 142 MJ, 39.4 kWh or 3.39 kg Oeq (HHV).

Assuming the total solar radiation of about 1500 kWh·m^−2^·yr^−1^ [10] and an energy conversion efficiency of 1%–3% (of total radiation), which will be discussed later, the energy produced as crude H_2_ is 15–45 kWh·m^−2^·yr^−1^ (Table 2). Assuming an energy recovery ratio (energy in purified H_2_/energy in crude H_2_) of 0.3–0.7, we calculated a total price for H_2_ of 10–40 ¢/kWh (*cf.* Section 1.3 for the price ranges of renewable fuels). Table 2 gives sales projections for photobiologically produced H_2_ and the energy conversion efficiency targets that will promote the adoption of photobiological renewable production.

### 4.1. Flexible Plastic Bioreactors Floating on the Surface of Sea Could Reduce Costs

Bioreactors for commercially viable algal biomass or liquid fuel production should be large (a total of a few km^2^ as a production unit) and inexpensive. Currently, open ponds with raceway configurations are the leading candidates for the first step of biomass production of algae. For H_2_ production, closed bioreactors are required in at least some part of the process. A variety of closed hard panel or tubular photobioreactors are described for laboratory or pilot-scale studies (e.g., [45,46]), but they are likely much more expensive than open ponds.

We have proposed flexible H_2_-barrier plastic bags floating on the calm sea or ocean surface (e.g., the calm belt about 30° north or south, such as the “mysterious Bermuda triangle” region) [17,44]. The culture medium is based on freshwater, and the bioreactors would spread over the sea surface because of the difference in density between the culture medium and the surrounding seawater. Salt lakes can also be used as the fields for cultivation. The size of the bags is flexible; for large-scale H_2_ production for example, 20 bags of 25 m × 200 m in surface area, could cover the surface of 1 km^2^. As proof of this concept, Kitashima *et al.* [47] demonstrated that transparent flexible H_2_-barrier plastic bags are usable for studies of photobiological H_2_ production by cyanobacteria in the laboratory. The H_2_ permeability (P_m_) value of the transparent plastic bags ranged from 44 to 87 cm^3^·m^−2^·atm^−1^·day^−1^. H_2_-barrier bags for laboratory use (Wakhy bags, www.ab.auone-net.jp/~wakhylab) are now commercially available (GL Sciences, Tokyo. info@gls.co.jp).

The cost of the bioreactor was estimated as follows [44]. The bioreactor is composed of three layers of plastic bags (Figure 3), for a total of six layers (sunny side and shady side) of transparent plastic film. The innermost bag holds the cyanobacteria culture, the middle bag has very low permeability to H_2_, and the outermost bag serves to mechanically protect the inner bags. The thickness of each film is 0.08 mm, and 480 cm^3^ of plastic per·m^2^ of the bioreactor’s sunny side surface is required. Assuming an average plastic price of $2–$4 per kg (or liter), the material cost is 96–192 cents·per·m^2^ of bioreactor. The used plastic bags can be recycled many times to regenerate plastic films at about half the price of the new materials. The above assumptions result in the cost of the bioreactor being about 24–48 cents per·m^2^ of bioreactor surface per year, assuming a renewal cycle of every two years. The plastic films of 480 cm^3^ are assumed to be produced by consuming 360 mL of crude oil for processing that is equivalent to 3.92 kWh·per·m^2^ per year. The plastic bags can be recycled at an energy cost of 20% of the feedstocks (0.78 kWh). The amount of energy in feedstocks derived from fossil fuels can be further decreased because currently H_2_ generated from fossil fuels is used as a part of the feedstocks for plastic film production, and photobiologically-produced H_2_ can replace some part of it.

### 4.2. Cost-Effective Strategy for Achieving Adequate Cell Growth with Repeated Harvesting of H_2_ without Changing the Culture Medium

Under our proposed strategy, H_2_ase-less mutant cyanobacterial cells grow in large ordinary plastic bags containing CO_2_ (with its occasional replenishment) floating on the sea without mechanical agitation of the medium (Step 1, in Figure 3). The need for additional combined nitrogen for growth would be minimal and thus the cost of fertilizer would be much less as compared to systems using non-nitrogen-fixing microalgae. Cyanobacterial stock cultures are transferred to photobioreactors for H_2_ production (Step 2). The innermost bag (Figure 3) is filled with gas containing CO_2_ in Ar. In the H_2_ production stage, no mechanical agitation or bubbling are applied, as CO_2_ is recycled within the bag (absorbed during photosynthetic carbon assimilation in vegetative cells and released from heterocysts when saccharides are degraded as electron donors to N_2_ase (Figure 2)). The cyanobacterial cell waste can be recycled as fish feed [48].

The proposed strategy can be modified in response to changes in production costs. For example, when the cost of Ar is high, N_2_ase mutants can be used [49]. Some of the mutants have high H_2_ production activity under N_2_-rich gas as compared to the parent strain activity under Ar gas, and also have extremely low N_2_-fixing activity. Examples of such mutants have been characterized; with the amino acid substitutions presumed to be located in the vicinity of the FeMo-cofactor of N_2_ase (e.g., Q193S and R284H) [49]. The N_2_ase activity of such mutants is not inhibited by the presence of high concentrations of N_2_ and thus the use of costly Ar could be avoided. However, these strains require combined nitrogen in the growth stage, and the advantages and disadvantages of using this type of mutant needs to be carefully assessed.

### 4.3. Initial Separation of crude H_2_ Followed by Further Purification by Pressure-Swing Adsorption (PSA) on Factory Ships

The H_2_ produced within the bag is allowed to accumulate for several weeks to a few months before “harvesting” the gas mixture (Figure 4). After removal of O_2_ from the harvested gas mixture, H_2_ is purified on factory ships by PSA (pressure-swing adsorption) for transportation to ports [44]. The separated Ar and CO_2_ are recycled back to the bioreactors, along with replenishment of H_2_O (substrate necessary for H_2_ production) to resume the next round of H_2_ production. The amount of H_2_O needed is relatively small, (0.8 kg H_2_O is required for 1 m^3^·H_2_, or 18 g for 22.4 L·H_2_). Some of the downstream technologies outlined in Figure 4 are still in the developmental stage, notably the initial separation of H_2_ from O_2_ in the gas mixtures. One of the candidate technologies involves the use of gas-selective membranes that allow the penetration of H_2_ at much higher rates than that of O_2_. For example, there is a report of a carbon molecular sieve membrane that allows permeation of H_2_ about 10–15 times faster than O_2_ (the gas permeability constant of 372–473 and 25–50 in gas permeability units for H_2_ and O_2_, respectively) [50]. We expect that there will be a concomitant development of these technologies if photobiological H_2_ production on the sea surface is adopted as one of the best options for large-scale renewable energy production (an example of “necessity is the mother of invention”).

The purified H_2_ may be condensed (high pressure or liquefied) or chemically transformed (e.g., to NH_3_ which can subsequently be used as a fuel rather than fertilizer, or reversible hydrogenation of toluene (C_7_H_8_) to methyl cyclohexane (C_7_H_14_)) for transportation to end-users (Figure 4).

### 4.4. The Advantages of Sustained High H_2_ Production without Changing the Culture Medium

We would like to emphasize that one of the economic advantages of photobiological H_2_ production by cyanobacteria is that the culture medium can be used continuously. During the growth stage, cyanobacteria do not produce H_2_ and the input costs of labor, energy, nutrients, *etc.* are minimal*.* A major advantage that the N_2_ase-based photobiological H_2_ production by cyanobacteria systems have over the other types of algal biofuel production systems is that once established, cultures can produce H_2_ for a long time (several weeks) without changing the culture medium and with multiple rounds of gas harvesting [49,51,52] (Table 3), thus off-setting the initial costs of the cell growth phase. Several groups have reported the duration of active H_2_ production to be several weeks, and we expect that this could be further extended by carefully managing the culture conditions (Table 3). Attaining sustained high H_2_ production without changing the culture medium will mitigate costs of nutrients, a criticism raised by Clarens *et al.* [42] (see Section 3.2).

**Table 3:** Some records of duration (>2 day) of H_2_ production activity without changing the culture medium.

Strains	Light Intensity	Efficiency (%)	Light Energy	Duration of expt.	Gas	Remarks	Refs.
*Anabaena cylindrica* 629	4.0 × 10^5^ erg·cm^−2^·s^−1^	0.4% av.	total rad.	8 d	Ar/CO_2_ (99.7/0.3), gassing	250 mL culture: about 0.6 L gas/h Cell density of 200–260 Klett units Periodic addition of 10^−4^ M·NH_4_Cl	[53]
*Anabaena cylindrica* 629	32 W·m^−2^	0.85% max.	PAR	6 d	Ar/CO_2_ (99.5/0.5), gassing	0.875 L culture: 0.3–0.9 L gas/h	[54]
*Anabaena cylindrica* 629	6 W·m^−2^	0.35% av.	PAR	28 d	Ar/CO_2_ (99.5/0.5), gassing	0.875 L culture: 0.3–0.9 L gas/h	[54]
*Mastigocladus laminosus* NZ-86-m	3.0 × 10^4^ erg·cm^−2^·s^−1^	2.7% max.	PAR	2–3 d	Ar/N_2_/CO_2_ (98.5/1/0.5), gassing	1.2 L culture	[55]
*Nostoc* sp. PCC 7422 ΔHup deficient of Hup	70 μmol photons m^−2^·s^−1^	3.7% av.	PAR	6 d	Ar/CO_2_ (9.5/5), (no gassing)	6 mL batch culture	[35]
*Anabaena variabilis* ATCC 29413	32 W·m^−2^	0.96% av. 1.32% max.	PAR	8 d	Stage 1: Ar, gassing, Stage 2: air/CO_2_ (95/5), gassing	H_2_ producing activity in stage 2	[56]
*Nostoc/Anabaena* sp. PCC 7120ΔHup, site-directed N_2_ase variants, Q193S and R284H	90–100 μmol photons·m^−2^·s^−1^	not given	PAR	21 d	N_2_/CO_2_ (95/5), (no gassing with periodic gas replacement of every 3 or 4 days)	Sustained, high-level production of H_2_ through 21 days	[49]

## 5. Improvements Needed in the Energy Conversion Efficiency under Outdoor Conditions

If photobiological H_2_ production is to be developed as a source of large-scale renewable energy, improvements are needed in both the light energy conversion efficiency and in the rates of H_2_ production. The maximum theoretical energy conversion for N_2_ase-based photoautotrophic H_2_ production by cyanobacteria has yet to be precisely calculated. This is because N_2_ase requires a considerable amount of ATP and the energy conversion efficiency of photophosphorylation has yet to be conclusively determined. There is a lack of information on the structure and function of the membrane ATP synthase (H^+^-ATPase) that consumes the proton-motive force generated across membranes as the source of energy as predicted by the chemi-osmotic mechanism proposed by P. Mitchell [57,58]. Most likely, the H^+^/ATP ranges between 8/3 and 14/3 (for discussion, see [21,59,60]). By assuming an H^+^/ATP ratio of between 3 and 4, Sakurai and Masukawa [17] calculated the maximum energy conversion efficiency of between 16.3% and 13.9% for 550 nm light and between 7.3% and 6.3% for the total solar radiation, assuming cyanobacteria can use 45% (400–700 nm light, PAR: photosynthetically active radiation) of total radiation.

Of special significance is a report of a record high H_2_ production activity on a chlorophyll basis by cyanobacteria. The N_2_ase activity of the unicellular cyanobacterium *Cyanothece* sp. ATCC 51142 is regulated by circadian rhythms: photosynthesis in the light, and N_2_ase reaction in the dark. When the cells were grown in the presence of glycerol for 12 h under the light to accumulate photosynthetic products (the first stage), they showed high photobiological H_2_ activity on a chlorophyll (Chl) basis of 465 μmol mg Chl^−1^·h^−1^ when the illumination was continued for another 12 h (the second stage) [61]. This type of strain may be potentially useful if low cost organics are available, such as wastewater rich in useable organic compounds (e.g., from the food industry). However, as many of the heterocystous strains very poorly use organic compounds, this is currently not very promising for the improvement of H_2_ production by heterocystous strains.

### 5.1. Comparing Measured Values for Light Energy Conversion Efficiency of Photobiological H_2_ Production by Cyanobacteria to Our Tentative Target Values of 1.2%

Although values of 2.6%–3.7% of PAR in laboratory experiments were reported by several groups [35,55,62], the values drop to 0.2% or less in outdoor experiments lasting for more than two days [51,52,63,64] (daily maximum of 0.6%, Miyamoto [51]) (Table 4). For our proposed scheme, we believe that 1.2% (of total radiation) or 2.7% of PAR for outdoor conditions are reasonable targets for improved efficiency.

**Table 4:** Some records of light energy conversion efficiency and duration of the activity.

Strains	Light Intensity	Efficiency (%)	Light Energy	Duration of expt.	Gas	Remarks	Refs.
*Mastigocladus laminosus* NZ-86-m	4.4 × 10^5^ erg·cm^−2^·s^−1^	0.17% av.	total rad.	17–24 d	Ar/N_2_/CO_2_ (98.7/1.0/0.3), gassing	1 L culture: 6.5 L·gas/h	[52]
*Anabaena cylindrica* 629	about 50–330 cal·cm^−2^·d^−1^	0.2% av. 0.6% max.	total rad.	36 d	Ar/N_2_/CO_2_ (balance/0.2–0.4/0.5), gassing	0.8 L culture: 5.0 L·gas/h	[51]
*Anabaena variabilis* PK84	400 W·m^−2^ (sunny day) 100 W·m^−2^ (cloudy day)	0.14% (sunny day) 0.33% (cloudy day)		18 d	air/CO_2_ (98/2), gassing	chemostat-type bioreactor, possibly V-type N_2_ase expressing conditions	[65]
*Anabaena variabilis* PK84 deficient of the activities of both Hup and Hox	max. 850 W·m^−2^	0.029%–0.094%	total rad.	40 d	air/CO_2_ (98/2), gassing	chemostat-type bioreactor, possibly V-type N_2_ase expressing conditions, June–July, London	[64]
*Anabaena* sp. PCC 7120 AMC 414 deficient of Hup	max. 600 W·m^−2^	0.042% max.	total rad.	9 d	air/CO_2_ (98/2), gassing	chemostat-type bioreactor, August, London	[63]

### 5.2. Some Potential Strategies to Achieve Higher Light Conversion Efficiencies

Many aspects of the H_2_ production and the related technologies are under development currently, making it challenging to estimate production costs. Nevertheless, such estimates are informative for both policy-makers and investors in R&D efforts. Sakurai *et al.* [44] presented a preliminary estimate of 26.4 ¢/kWh (assuming the price of plastics at $2 per kg) as a means to identify the needed R&D efforts to achieve efficient photobiological H_2_ production by cyanobacteria. The conditions were: (1) mariculture-raised cyanobacteria produce H_2_ at an energy conversion efficiency of 1.2% (18 kWh·m^−2^·yr^−1^) in plastic bioreactors floating on the sea surface (solar radiation 1500 kWh·m^−2^·yr^−1^); (2) the crude H_2_ is purified on factory ships and transported to final destination ports in storage tanks by container ships; and (3) the energy recovery (from production to the final commodity of H_2_) is 0.5. Although the tentatively estimated price above is more expensive than the currently levelized price of electricity, it is reasonably close to the prices in several countries with feed-in tariff schemes (see Section 1.3). The price of H_2_ can be further decreased with improvements in both light conversion efficiency and energy recovery, and by decreasing the processing costs with advancements in relevant technologies. Because photobiologically produced H_2_ contributes to the reduction of the greenhouse gas CO_2_ emissions, and because H_2_ fuel cells are expected to be more energy efficient than internal combustion engines, the R&D of photobiological renewable energy sources should be pursued in more earnest.

Amos [66] estimated the cost of H_2_ produced by *Chlamydomonas* to be 22.8 ¢/kWh when H_2_ is compressed and stored in high-pressure tanks for storage and transportation, and 7.2 ¢/kWh when direct connections to H_2_ pipelines are available. In certain locations, such as salt lakes, a pipeline could be used to transport gas products with reduction of the total cost of H_2_ photobiologically produced by cyanobacteria.

#### 5.2.1. Selection of Strains with High N_2_ase Activity Outdoors Followed by Further Improvements via Genetic Engineering

Yoshino *et al.* [35] compared the photobiologically driven N_2_ase activity under laboratory conditions among 13 heterocystous strains maintained in academic centers, and selected *Nostoc* sp. PCC 7422. The uptake hydrogenase knocked out mutant (∆Hup) produced H_2_ at an energy conversion efficiency of 3.7% *vs.* PAR under laboratory conditions. Selection of strains with high N_2_ase activity under outdoor conditions with high light intensities followed by further targeted improvements via genetic engineering seems to one of the most promising strategies for achieving higher light conversion efficiencies.

#### 5.2.2. Truncated Antenna Complexes

Light-saturation of photobiological activity is one of main reasons light conversion efficiencies are decreased under outdoor conditions as compared with the laboratory conditions for cyanobacteria. Many cyanobacterial strains have large antenna complexes containing pigments such as phycocyanin, which enable them to survive even under conditions of low light intensity such as in a dense culture or in sediment. In order for photobiological H_2_ production schemes of the culture as a whole to operate at optimum efficiency, large antenna size is a problem because cells near the surface absorb the most part of incident light and are unable to use it efficiently because of light saturation, while other cells beneath them receive only the residual low light. It is hypothesized that truncation of antenna size would help to alleviate excess absorption of sunlight and the ensuing wasteful dissipation of excitation energy, and to improve solar-to-product energy conversion efficiency and photosynthetic productivity in high-density mass cultivations. As proof of this concept, there are reports of truncated antenna cyanobacterial mutants showing higher rates of photosynthetic activity in culture over the wild-type strains [67].

#### 5.2.3. Increase in Heterocyst Frequency

Under conditions of nitrogen deprivation, heterocystous cyanobacterial cells differentiate heterocysts at intervals of 10–20 vegetative cells, depending upon the strains. While heterocyst frequency may be optimized to support growth of the strains while fixing N_2_ under their ordinary natural habitats, the frequency can also be modified to increase H_2_ production because the cells do not grow in the H_2_ production stage and thus the supply of organic compounds for growth can be dispensed. Many genes involved in heterocyst differentiation have been reported and the mutants of several of these genes (via disruption, overexpression, and duplication of genes and point mutation) result in higher frequencies of heterocysts [25,26,68]. However, many of the mutants form multiple contiguous heterocysts and their N_2_ase activities measured by acetylene reduction do not exceed the wild-type activities. PatN is required for normal heterocyst patterning and has a role in the biased initiation of heterocyst differentiation. In *Nostoc*/*Anabaen*a sp. PCC 7120, disruption of *patN* led to formation of multiple singular heterocysts. Although the *patN* mutant exhibited lower N_2_ase activity and diazotrophic growth rate compared with the wild-type [69], it will be interesting to see the effects of modification of heterocyst frequency on photobiological H_2_ production by other strains or under different experimental conditions.

#### 5.2.4. Improvement of the Enzymatic Activity of N_2_ase

Other H_2_-producing enzymes, FeFe and NiFe H_2_ases, have high H_2_ production activity with a turnover rate of 6000–9000 and about 100 s^−1^, respectively, although the latter enzyme also has greater H_2_ uptake activity with a turnover rate of 450–600 s^−1^ [70]. In contrast, although N_2_ase has an advantage in catalyzing unidirectional production of H_2_, it has a very low turnover rate of about 6.4 s^−1^ [71,72]. The rate-limiting step in N_2_ase catalysis is the dissociation of the dinitrogenase reductase Fe protein (Complex 2) from the dinitrogenase MoFe protein (Complex 1). The dissociation occurs after ATP-coupled electron transfer between the two proteins. In the N_2_ase reaction, H_2_ is evolved initially when N_2_ binds to the enzyme. The slow protein dissociation is considered to contribute to maximizing N_2_ reduction by suppressing H_2_ evolution, futile to nitrogen fixation, at the midpoint of the whole reaction. It will be interesting to engineer N_2_ase, either MoFe-protein or Fe-protein, or both, by site-directed mutagenesis, to increase the turnover rate of H_2_ production by the enzyme.

When maintaining the N_2_ase-based reactions at high levels, cyanobacteria require high levels of amino acids to support the synthesis of the inefficient enzyme, and the increase in the specific catalytic activity will decrease the burden of protein synthesis.

#### 5.2.5. Metabolic Engineering

As in other organisms, studies of genomes, transcriptomes, proteomes, and metabolomes are ongoing in cyanobacteria. The information from these studies will undoubtedly contribute to improving the overall activity of photobiological H_2_ production by cyanobacteria, as targeted improvements will be made [73]. For example, it will be interesting to pursue repression of the expression of glutamine synthetase in heterocysts. In normal cells, the enzyme is required for the massive export of fixed nitrogen from heterocyst to vegetative cells, and the gene expression level is greatly increased during the course of heterocyst differentiation [74]. In H_2_ producing cyanobacteria, however, massive glutamine transport is not necessary because the cells almost cease growing, and the investment of amino acids for the synthesis of proteins such as glutamine synthetase seems to be a waste of amino acid reserves.

## 6. Conclusions

### Future Prospects for Large-Scale Photobiological H_2_ Production

In order for photobiologically produced H_2_ to make meaningful contributions to the mitigation of global warming caused by greenhouse gases, notably CO_2_, economical production of H_2_ is essential. Although many of the technologies required for the practical application of large-scale photobiological H_2_ production are in the development stages and the cost estimates of the produced H_2_ are preliminary due to various unpredictable factors, Sakurai *et al.* [44] presented a very preliminary estimate of 26.4 cents·kWh^−1^ of H_2_ produced on the sea surface using many presumptive assumptions, for example assuming the solar energy conversion efficiency of 1.2%. Currently, the maximum energy conversion efficiency of cyanobacterial H_2_ production under outdoor conditions is about 0.2%. There is an urgent need to demonstrate higher efficiencies to policy makers and developers in order to convince them of the potential benefits of photobiological H_2_ production by cyanobacteria. Increasing the solar energy conversion efficiency with improvements in technologies for purifying and transporting H_2_ will decrease the total cost. Public acceptance of renewable fuels very much depends upon the affordability of the price and will go a long way towards convincing consumers and government leaders to adopt technologies that will ultimately mitigate global climate change. Future cost estimates will be more accurate as the technologies in cyanobacterial H_2_ production and its related processes advance. We also expect that more refined cost estimates will allow for additional targeted improvements in the technologies, ultimately resulting in an overall cost reduction.

If we are able to produce H_2_ at 1.2% of total radiation (18 kWh·m^−2^·yr^−1^) on the sea surface and purified H_2_ is delivered to the end users with a final energy yield of 0.5, the net energy obtained is calculated to be 9 kWh·m^−2^·yr^−1^ or 32.4 × 10^6^ J·m^−2^·yr^−1^ [44]. The current world fuel energy consumption is estimated to be 460 × 10^18^ J·yr^−1^ (Table 1, [3]). It follows that by using a surface of the sea equivalent to 1% of the global surface (1.36 × 10^6^ km^2^, about 2.3 times the area of the island of Tasmania, or 3.1 times the area of the island of Great Britain), we will be able to replace 19% of the current world fossil fuel consumption.

In order to make a meaningful contribution to the mitigation of the hazards to the human population that are predicted to occur with global climate change, the promotion of R&D efforts for economical large-scale photobiological biofuel production and the advancements of biological and other needed technologies should be encouraged.
