# Correction: Sakurai, H.; et al. How Close We Are to Achieving Commercially Viable Large-Scale Photobiological Hydrogen Production by Cyanobacteria: A Review of the Biological Aspects. *Life* 2015, *5*, 997–1018

**DOI:** 10.3390/life8040048

**Published:** 2018-10-19

**Authors:** Hidehiro Sakurai, Hajime Masukawa, Masaharu Kitashima, Kazuhito Inoue

**Affiliations:** 1 Research Institute for Photobiological Hydrogen Production, Kanagawa University, Tsuchiya, Hiratsuka, Kanagawa 259-1293, Japan; inouek01@kanagawa-u.ac.jp; 2 The OCU Advanced Research Institute for Natural Science and Technology (OCARINA), Osaka City University, 3-3-138 Sugimoto, Sumiyoshi-ku, Osaka 558-8585, Japan; jimimasu@gmail.com; 3 Department of Biological Sciences, Kanagawa University, Tsuchiya, Hiratsuka, Kanagawa 259-1293, Japan; pt125536zy@kanagawa-u.ac.jp

In the published article “How close we are to achieving commercially viable large-scale photobiological hydrogen production by cyanobacteria: A review of the biological aspects”, *Life* 5, Page 999, line 9–10 [1]:

fuel-derived energy) (Table 1): people in US 136, Germany 76, Japan 70, China 43 times, respectively (IEA Energy Indicators 2012 in (IEA Energy Outlook 2014))

Should read as:

fuel-derived energy) (Table 1): people in US 100, Germany 56, Japan 52, China 31 times, respectively (IEA Energy Indicators 2012 in (IEA Key World Energy Statistics 2014)).

The changes do not greatly affect the point of the main argument, and we would like to apologize the inconvenience this has caused. The manuscript will be updated and the original will remain online on the article webpage with this Correction.
